# Comparison of the types of candidate reference samples for quality control of human epidermal growth factor receptor 2 status detection

**DOI:** 10.1186/s13000-016-0537-8

**Published:** 2016-09-10

**Authors:** Yulong Li, Rui Zhang, Yanxi Han, Tian Lu, Jiansheng Ding, Kuo Zhang, Guigao Lin, Jiehong Xie, Jinming Li

**Affiliations:** 1 National Center for Clinical Laboratories, Beijing Hospital, National Center of Gerontology, No1 Dahua Road, Dongdan, Beijing, 100730 People’s Republic of China; 2 Graduate School, Peking Union Medical College, Chinese Academy of Medical Sciences, Beijing, People’s Republic of China; 3 Beijing Engineering Research Center of Laboratory Medicine, Beijing Hospital, National Center of Gerontology, Beijing, 100730 People’s Republic of China

**Keywords:** Quality control, Cell lines, Agarose gel, Xenograft, Clinical specimens, FISH, IHC

## Abstract

**Background:**

Human epidermal growth factor receptor 2 (*HER2*) is as a target gene for trastuzumab in patients with breast cancer. Accurate determination of HER2 status and strict quality control are necessary to ensure reproducibility and accuracy of the techniques used for the determination of HER2 status.

**Methods:**

We used three different types of samples: formalin-fixed and paraffin-embedded (FFPE) samples prepared from cell lines, agarose gel samples using cell lines, and xenograft tumor samples. One cell line for FFPE or xenografts did not overexpress HER2, while the others showed different levels of HER2 overexpression. We compared the morphology, *HER2* gene amplification status, and HER2 protein expression status of these samples with those of clinical specimens.

**Results:**

We successfully produced three kinds of samples for quality control. Cells from the cell line-sample sections were dispersed while those from the agarose gel-sample sections and xenograft tumor sample sections (prepared from the both cell lines) were concentrated in one area. The FISH results for all three kinds of samples were as expected. The IHC results of the cell line samples and xenograft tumor samples were as expected, but the staining level of the agarose gel samples, using HER2-overexpressed cell lines was weak which might be regarded as a false negative result.

**Conclusions:**

Xenograft tumor samples might be used as an additional option for quality control in FISH and IHC. However, it might not replace the clinical specimen quality controls directly.

**Electronic supplementary material:**

The online version of this article (doi:10.1186/s13000-016-0537-8) contains supplementary material, which is available to authorized users.

## Background

Approximately 25 % of patients with invasive breast cancer overexpress human epidermal growth factor receptor 2 (HER2) [1]. HER2 overexpression, mainly because of *HER2* amplification, is significantly associated with aggressive disease and poor prognosis [2]. Trastuzumab can bind the extracellular domain of HER2, and has a remarkable impact on the treatment of patients with HER2-positive breast by several pathways [3–5]. Therefore, trastuzumab was approved by the United States (US) Food and Drug Administration (FDA) for the treatment of breast cancer. Hence, accurate determination of the HER2 status in patients with breast cancer is crucial [6].

Immunohistochemistry (IHC), for measuring protein overexpression, and fluorescence *in situ* hybridization (FISH), for measuring *HER2* amplification, are the most commonly applied methods for the assessment of HER2 status [7]. Although both these methods can be applied to evaluate protein overexpression and gene amplification in relation to the morphological features of tumors, there is large inter-laboratory variation in the procedure, staff, and interpretation, which affects the analytical sensitivity and specificity of these assays, especially in the case of indeterminate samples [8].

Ensuring the accuracy and reproducibility of HER2 detection by IHC and FISH testing is a fundamental prerequisite for targeted therapy. Recommendations for HER2 testing were developed by the American Society of Clinical Oncology (ASCO) and the College of American Pathologists (CAP) in 2007 and were updated in 2013 [9, 10] to improve the performance of these tests. HER2 detection requires precisely characterized and universally available reference controls [11]. Adequate quality control is necessary to diminish inter-laboratory variation and external quality assurance.

Recently, a consensus was reached to create clinical specimen or cell line controls as reference materials for HER2 testing [12]. Quality control of clinical specimens facilitates the assessment of variation in methodologies and laboratories. However, some disadvantages have limited the development of these quality controls, such as production and ethics. Therefore, we developed new xenograft tumor controls and checked their suitability for internal quality control (IQC) and external quality assurance (EQA). In this study, we compared three quality control samples with traditional clinical specimens. The aim of our study was to identify the most appropriate quality control that would ensure maximum accuracy and reproducibility of IHC and FISH.

## Methods

### Cell lines

The breast cancer cell lines MCF-7, MDA-MB-453, BT474, and SKBR-3 were obtained from the Cell Based Medical Center of Basic Medicine of Peking Union Medical College (Beijing, China). Cells were cultured as recommended by the suppliers. The HER2 status of the cell lines has been reported in previous studies [1, 13]. MDA-MB-453, BT474, and SKBR-3 expressed HER2, while MCF-7 did not. All four cell lines were applied for the preparation of three quality-control samples as described below.

### Samples

Two surgical specimen cases of invasive breast cancer with of known HER2 status were selected and obtained from the Pathology Department of Beijing Hospital, Beijing, China. Our protocol and specimen were approved by the Ethics Committee of the National Center for Clinical Laboratories. One of the samples tested negative for *HER2* (A2; FISH result: negative; IHC score: 0), whereas the other sample tested positive (A12; FISH result: positive; IHC score: 3+). The HER2 status of these two samples was evaluated together with our prepared samples.

### Preparation of formalin-fixed and paraffin-embedded (FFPE) cell line samples

Approximately 1 × 10^7^–2 × 10^7^ cells were obtained and fixed in 10 % neutral-buffered formalin for approximately 2 h at room temperature. Subsequently, cell pellets were processed with gradient ethanol for dehydration and with xylene for transparency. Cell pellets were treated with melted liquid paraffin in small Eppendorf tubes and waxed thoroughly for 10 min; liquid paraffin was mixed with the cell pellets, using pipette tips. The solution was then chilled to achieve solidification, and the resulting blocks were embedded in paraffin, using a standard histochemical apparatus. Hematoxylin-eosin (HE) staining was performed to preserve the representative morphology of each sample and the number of cells to assess the uniformity of cellular distribution for each section. Each section was 5 μm in thickness. The sections were mounted onto slides and dried in an oven at 70 °C for approximately 3 h to ensure maximum adhesion.

### Preparation of FFPE agarose gel samples

Approximately 1 × 10^6^-2 × 10^6^ cells were fixed overnight in 10 % neutral-buffered formalin at 4 °C, with the fixed cells being allowed to sediment at the base of a 15-mL tube with a conical base after centrifugation at 0.4 × *g* for 10 min, following which the formalin supernatant was removed. The cells were resuspended in phosphate buffered saline (PBS) solution, and the suspension was transferred to 1.5-mL Eppendorf tubes and allowed to sediment again after centrifugation at 0.4 × *g* for 10 min. The supernatant was removed, leaving the cells in 10–50 μL of PBS, in which the cells were resuspended. The cell suspension was embedded in prewarmed and equilibrated 3 % PBS-buffered agarose gel in 5-mm diameter × 5-mm deep iron molds from which small agarose-cell mixture cylinders were extruded after the agarose was sufficiently chilled. The agarose gel cylinders were then placed in a standard tissue-processing cassette and processed with paraffin wax. HE staining was performed; each section was 5 μm in thickness.

### Preparation of FFPE xenograft tumor samples

Female severe combined immunodeficient (SCID) mice between 21 and 28 days of age (Vital River, Beijing, China) were used; the mice were quarantined for at least 3 days prior to the study. The mice were anesthetized with vaporized isoflurane, and suspensions of cells (all the four cell lines, approximately 1 × 10^7^ cells) mixed with Matrigel™ basement membrane matrix (Corning, New York, USA) were injected subcutaneously under sterile conditions into the backs of mice, using a 5-gauge needle. Once the size of the xenograft tumor had progressed to approximately 500 mm^3^, the mice were euthanized via cervical dislocation, and all xenograft tumors belonging to different cell lines were removed surgically. All animal experiments were performed in compliance with the guidelines specified by the Institute for Experimental Animals, Beijing Hospital. Following surgical removal of xenograft tumors from the SCID mice, the tumors were cut into multiple pieces of approximately 250 mm^3^, placed into appropriately labeled disposable plastic tissue cassettes, and then immediately fixed in 10 % neutral-buffered formalin at 4 °C overnight. Subsequently, automated tissue embedding was performed using a Renaissance Tissue Processor (Ventana Medical Systems, US). This included washing in a graded ethanol series for dehydration followed by xylene washes for transparency, and heated paraffin washes prior to embedding in paraffin. HE staining was performed to ensure that the representative morphology of each sample was maintained. Each section was cut to a thickness of 5 μm.

### Evaluation of *HER2* status by FISH

FISH assays were performed following the manufacturers’ instructions (PathVysion HER-2 DNA Probe Kit, Abbott, Chicago, IL, USA). In brief, deparaffinization, heat pretreatment, and protease treatment were performed sequentially for all the FFPE samples. After pretreatment, the sample sections and probes were co-denatured and hybridized. A post-hybridization wash was performed for all sample sections in the dark using the wash buffer supplied by the manufacturer. 4′,6-Diamidine-2′-phenylindole dihydrochloride (DAPI) counter-stain was applied to the target area of the sections, and fluorescence microscopy was performed to view areas of positive hybridization. The results were evaluated qualitatively based on the kit recommendations.

### Evaluation of HER2 expression by IHC analysis

The HER2 protein expression status of the sample slides was assessed using a commercial primary antibody (PATHWAY® anti-HER-2/neu (4B5) Rabbit Monoclonal Primary Antibody; Ventana, Tucson, AZ, USA), as per manufacturer’s instructions. Briefly, all the sample slides were processed according to a standard tissue-processing process, including deparaffinization, rehydration and antigen retrieval. After cooling for 15 min, IHC reactions were manually performed manually using the primary monoclonal antibody, 2-step plus® Poly-HRP Anti Mouse/Rabbit IgG Detection System (ZSGB-BIO, Beijing, China) and diaminobenzidine (DAB) staining. HER2 protein expressed on the membrane of tumor cells was scored as 0+, 1+, 2+, or 3+ as recommended [10].

## Results

### Characteristics of the three types of samples for quality control

**Fig. 1:**
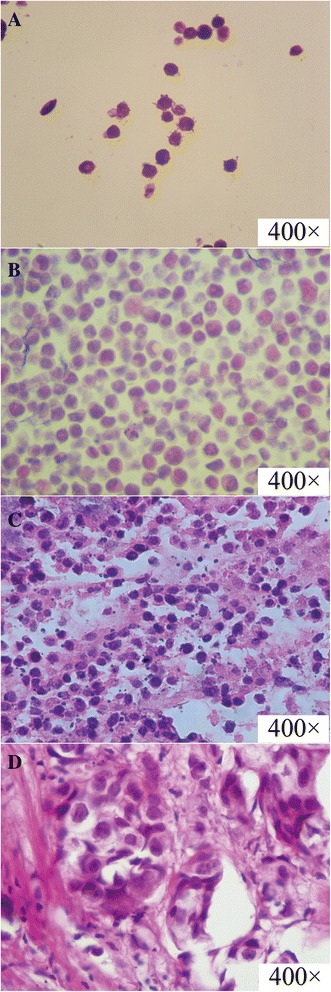
The HE staining results of three kinds of samples compared with clinical specimen. **a** is section of cell lines sample; **b** is section of agarose gel within cell lines sample; **c** is section of xenograft tumor sample. All these sections were derived from cell lines of MCF-7. **d** is section of clinical specimen (A2)

In this study, we successfully developed three types of samples that may be used for quality control in the determination of *HER2* status. The morphological features of these samples in comparison with those of clinical specimens are shown in Fig. 1. In brief, the target cells in the cell line samples were well dispersed, but cells embedded in the agarose gel samples were concentrated in one area. However, the xenograft tumor samples were similar to tumor tissue samples derived from patients with breast cancer. Nests of carcinoma were found in both the xenograft tumor samples and clinical specimens. However, some distinctions were also found in the samples. In the clinical specimens, a sizeable number of normal cells was found, and the tissue structure was complicated. Apart from the nests of carcinoma, breast catheter and breast glands were observed. Inflammatory cell infiltration was evident around the nests of carcinoma. In the xenograft tumor samples, almost all the cells were tumor cells, and the tissue structure was simple. Apart from the target tumor cells, only red blood cells, fibroblast cells, and vascular endothelial cells were detected. The other characteristics of the samples, as well as the technology used and costs are shown in Table 1.

**Table 1:** The characteristic of three samples for quality control

Quality control samples	Cell lines samples	Agarose gel samples	Xenograft tumor samples	Clinical specimens
Morphological feature	Diffused distribution without tissue structure	Concentrated distribution without tissue structure	Concentrated distribution with simple tissue structure	Concentrated distribution with complicated tissue structure
Target cells count in each section	>10^4^	>10^3^	Countless	>10^2^
Adhesion characteristic	Bad	Good	Good	Bad
Production process	Easy	Easy	Difficult	Difficult
Production period	1 month	1 month	3–4 months	About 2–4 months [15]
Production cost (£/sample on one section)	2.03	2.07	2.22	About 10 [15]

### Evaluation of *HER2* status by FISH

**Fig. 2:**
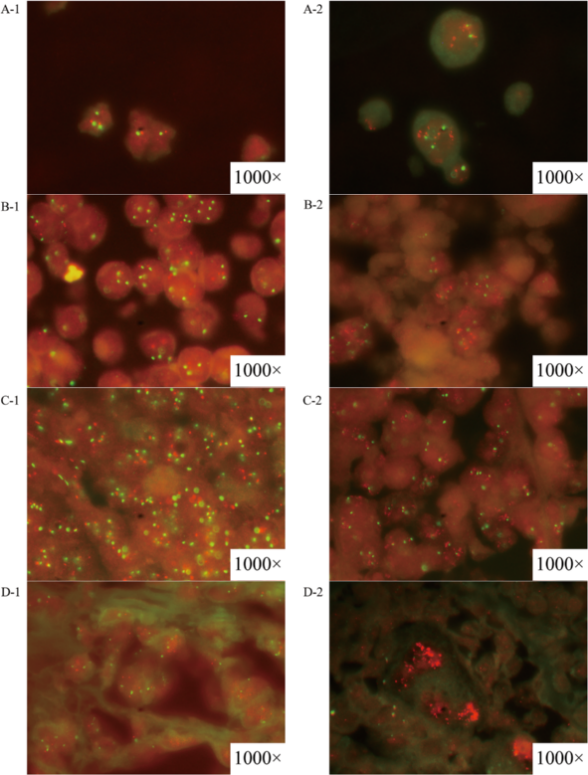
The results of HER2 gene amplification status evaluated by FISH assay. A-1 was section of cell lines sample without HER2 gene amplification (MCF-7) while A-2 was that with positive HER2 gene amplification (SKBR-3). B-1 was section of agarose gel within cell lines sample without HER2 gene amplification (MCF-7) while A-2 was that with positive HER2 gene amplification (SKBR-3). C-1 was section of xenograft tumor sample without HER2 gene amplification (MCF-7) while A-2 was that with positive HER2 gene amplification (SKBR-3). D-1 was section of clinical specimen without HER2 gene amplification (A2) while A-2 was that with positive HER2 gene amplification (A12). The magnification was 1000 power

All samples prepared using the MCF-7 cell line did not show *HER2* amplification. All samples prepared MDA-MB-453, BT474, and SKBR-3 showed positive results, and samples using BT474 and SKBR-3 showed highly positive results, with a cluster of positive signals (Table 2 and Additional file 1). Among the clinical specimens (Table 2 and Fig. 2), A2 was negative and A12 was highly positive for *HER2* amplification. Together, these results suggested that all the samples could be applied for quality control in FISH assays.

**Table 2:** The results of FISH and IHC method to evaluate three samples made of different cell lines for quality control

Quality control samples	Cell lines’ name or patient’s code	The results of FISH detection			The results of IHC detection	
		Qualitative results	Ratio of HER2:chromosome 17	HER2 copy number	Qualitative results	IHC scoring
Cell lines samples	MCF-7	Negative	1.15	3.05	Negative	0
	MDA-MB-453	Positive	2.26	6.9	Positive	3+
	SKBR-3	Positive	Clusters	Clusters	Positive	3+
	BT474	Positive	Clusters	Clusters	Positive	3+
Agarose gel samples	MCF-7	Negative	1.18	3.85	Negative	0
	MDA-MB-453	Positive	2.15	7.1	Negative	0
	SKBR-3	Positive	Clusters	Clusters	Negative	1+
	BT474	Positive	Clusters	Clusters	Positive	2+
Xenograft tumor samples	MCF-7	Negative	1.27	5.25	Negative	0
	MDA-MB-453	Positive	2.42	8	Positive	2+
	SKBR-3	Positive	Clusters	Clusters	Positive	3+
	BT474	Positive	Clusters	Clusters	Positive	3+
Clinical specimens	A2	Negative	1.07	4.07	Negative	0
	A12	Positive	Clusters	Clusters	Positive	3+

### Evaluation of HER2 expression by IHC analysis

**Fig. 3:**
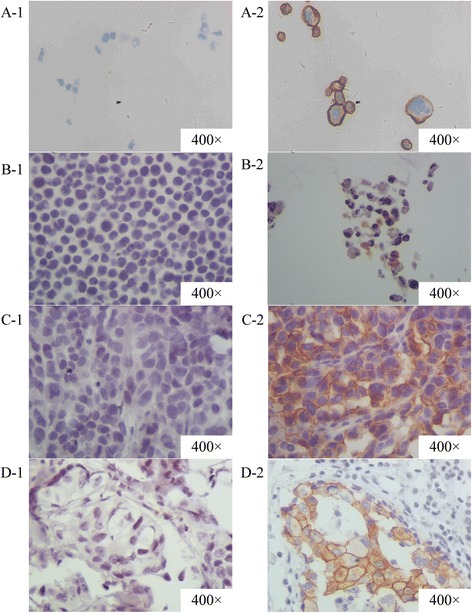
The results of HER2 protein expression status evaluated by IHC assay. A-1 was section of cell lines sample without HER2 overexpression (MCF-7) while A-2 was that with positive HER2 overexpression (SKBR-3). B-1 was section of agarose gel within cell lines sample without HER2 overexpression (MCF-7) while B-2 was that with positive HER2 overexpression (SKBR-3). C-1 was section of xenograft tumor sample without HER2 overexpression (MCF-7) while C-2 was that with positive HER2 overexpression (SKBR-3). D-1 was section of clinical specimen without HER2 overexpression (A2) while D-2 was that with positive HER2 overexpression (A12). The magnification was 400 power

A comparison of HER2 expression between the samples developed by us and the clinical specimens is shown in Table 2 and Fig. 3. Cell line samples and xenograft tumor samples prepared from SKBR-3, BT474, and MDA-MB-453 showed overexpression of HER2 protein localized to the cell membrane (IHC score: 3+) relative to samples prepared using MCF-7 (lower expression; IHC score: 0) and the negative clinical sample (A2). In addition, cell line samples and xenograft tumor samples using SKBR-3 and BT474 expressed higher levels of HER2 than samples prepared using MDA-MB-453 and the positive clinical sample (A12). There was no difference of staining between the cell line samples and their corresponding xenograft tumor samples. However, agarose gel samples prepared using MCF-7, MDA-MB-453, and SKBR-3 showed negative results, and the HER2 expression in agarose gel samples prepared using BT474 was lower than that in the cell line samples and xenograft tumor samples prepared using BT474. The detailed IHC staining scores are shown in Table 2 and Additional file 2.

## Discussion

Quality controls are required in HER2 status detection to ensure the accuracy and reproducibility of testing [2, 10] and hence, choosing a suitable quality control is necessary for FISH and IHC assays. Currently, the best quality controls for HER2 testing are tissue-based controls from clinical specimens exhibiting both HER2 overexpression and *HER2* amplification that have been confirmed by IHC and FISH [2]. Some studies have also reported that cell line samples can be used for EQA as additional quality controls instead of traditional quality controls derived from patient tumor tissue [13, 14]. In this study, we have developed three quality controls and compared them with cell line samples and clinical specimens to identify a suitable quality control for FISH and IHC assays.

Different quality controls possess characteristic advantages and disadvantages. At present, tissue microarray (TMA)-based or FFPE-based quality controls prepared from clinical specimens are widely used for EQA or IQC in IHC or FISH testing [15, 16]. The protocol utilizing clinical specimen quality controls is robust, cost-effective, and rapid. Moreover, it allows continuous monitoring of the performance of FISH and IHC testing [16]. However, some potential limitations of clinical specimen quality controls should be considered. Since, EQA requires a larger number of quality controls, a collection of large clinical specimens from different patients with breast cancer is needed. Previous studies have reported that the properties of the samples being tested may influence the results and create bias in the comparison [17]. Thus, heterogeneity among clinical specimens from different patients may affect the reproducibility of assays. Moreover, mass production of clinical specimens is difficult, and ethical problems should be taken into consideration. As additional quality controls, cell line quality controls are currently applied for IQC and EQA instead of traditional clinical specimens. Compared with traditional paraffin blocks of tumor tissues, large amounts of such controls can be obtained easily, and the reproducibility allos for sensitive detection and decreases analytical errors [11]. In our study, we found that cell line samples can be applied for both IHC and FISH, as reported by previous studies [11, 18], and that the protein and gene status of different cell lines can be determined accurately.

However, we found two main disadvantages in the use of cell line quality controls. First, the tumor cells showed diffuse distribution in the entire section, and the diffused cells were difficult to observe and count. The diffused cells were also prone to drop off for lack of tissue structure. Second, cultured cells were prone to polysomy, which is uncommon in patient tumor tissue. This polysomy might affect the reproducibility of cell line quality controls [19].

To resolve these issues, agarose gel samples prepared using different cell lines were produced to optimize the basic cell line samples. Embedding in agarose gel helped gather the cells and increase the viscosity of the specimen. However, the morphological features of actual human tumors could not be simulated. Furthermore, we found that the agarose gel samples could only be applied for FISH. The use of an overheated agarose gel led to changes in the morphology of cells. Additionally, the use of agarose gel affected the positive results because of weak or faded staining. The probable reason for this is the effect of fixation or storage on the agarose gel, which has been previously reported to decrease the staining in IHC [20]. Thus, agarose gel samples were not suitable as quality controls.

On the basis of the results of this study, we suggest xenograft tumor samples as suitable additional quality controls instead of cell line quality controls. Our observations indicate that xenograft tumor samples are similar to patient tumor specimens, providing evidence that they can be implemented for quality control. A previous study, which applied xenograft tumor samples as the quality control in the IHC assessment of estrogen receptor (ER) and progesterone receptor (PR), reported that the use of xenograft tumor samples as quality controls provides advantages in relation to reproducibility of biomarker, homogeneity of tissue histology, specimen processing, availability, and cost compared with archived tumor quality controls and cell line quality controls [21]. Similarly, our study confirmed that xenograft tumor samples could be applied for quality control in the detection of HER2 status. Furthermore, our study proved that xenograft tumor quality controls could be applied for the detection of both protein status (IHC) and gene status (FISH). Regarding the economic factor, the cost of producing xenograft tumor samples is similar to that involved in producing cell line samples but lesser than that involved in preparing clinical specimen tissue TMA or FFPE-based quality controls (Table 1) [15].

However, some potential disadvantages need to be considered. The characteristic features of cell lines for establishing xenografts should be monitored during long-term culture to ensure reliability. In theory, although the target tumor cells in xenografts are human-derived [22], they might contain vessels or fibrocytes derived from mice. This indicates that the non-specificity of the secondary antibody might lead to some background staining in IHC. However, in our study, we found that the influence of this background staining was negligible. Nevertheless, poor tumorigenic potential and slow growth of HER2-positive xenograft tumors might impede its application [23]. In our study, we chose SCID mice and Matrigel basement membrane matrix to improve the tumorigenic potential [24]. Moreover, some distinctions were found between the xenograft tumor samples and clinical specimens in histological analysis. In the clinical specimens, a larger number of normal cells was observed and the tissue structure was more complicated. Breast catheter, breast gland, and inflammatory cell infiltration was evident. In the xenograft tumor samples, almost all the cells were tumor cells, and the tissue structure was simple. As a result, the evaluation of clinical specimens was more difficult than in the case of xenograft tumor samples. Some performance characteristics, especially the interpretation by observers in the routine work, might be neglected to be evaluated in EQA.

## Conclusions

In this study, we compared three types of candidate reference samples with clinical specimens for quality control of HER2 status detection. The study indicated that xenograft tumor samples might be more suitable for FISH and IHC as additional alternative quality controls than cell line quality controls. However, xenograft tumor samples cannot replace the traditional clinical specimen quality controls until issues such as tumorigenic potential and distinctions in histology are resolved.

## Additional files

Additional file 1: The detailed results of HER2 gene amplification status evaluated by FISH assay. A-1 to A-4 were section of cell lines samples which were derived from MCF-7, MDA-MB-453, SKBR-3, BT474, respectively. B-1 to B-4 were section of agarose gel within cell lines samples which were derived from MCF-7, MDA-MB-453, SKBR-3, BT474, respectively. C-1 to C-4 were section of xenograft tumor samples which were derived from MCF-7, MDA-MB-453, SKBR-3, BT474, respectively. The magnification was 1000 power. (PDF 17214 kb) Additional file 2: The detailed results of HER2 protein expression status evaluated by IHC assay. A-1 to A-4 were section of cell lines samples which were derived from MCF-7, MDA-MB-453, SKBR-3, BT474, respectively. B-1 to B-4 were section of agarose gel within cell lines samples which were derived from MCF-7, MDA-MB-453, SKBR-3, BT474, respectively. C-1 to C-4 were section of xenograft tumor samples which were derived from MCF-7, MDA-MB-453, SKBR-3, BT474, respectively. The magnification was 400 power. (PDF 17387 kb)

## Abbreviations

ASCO: American Society of Clinical Oncology
CAP: College of American Pathologists
DAB: Diaminobenzidine
DAPI: 4′,6-Diamidine-2′-phenylindole dihydrochloride
EQA: External quality assurance
ER: Estrogen receptor
FDA: Food and Drug Administration
FFPE: Formalin-fixed and paraffin-embedded
FISH: Fluorescence in situ hybridization
HE: Hematoxylin-eosin
HER2: Human epidermal growth factor receptor 2
IHC: Immunohistochemistry
IQC: Internal quality control
PBS: Phosphate buffered saline
PR: Progesterone receptor
SCID: Severe combined immunodeficient
TMA: Tissue microarray
US: The United States
