# Supplementary figures and images for: Comparison of the types of candidate reference samples for quality control of human epidermal growth factor receptor 2 status detection
Source: Diagn Pathol. 2016 Sep 10;11(1):85. doi: 10.1186/s13000-016-0537-8 (PMC5018185; doi:10.1186/s13000-016-0537-8)

A-1

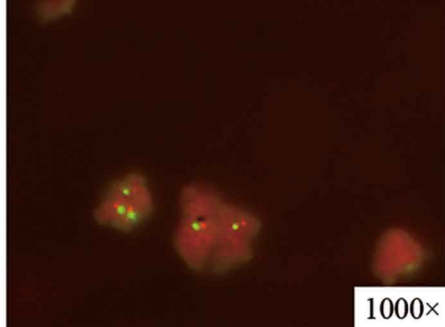

B-1

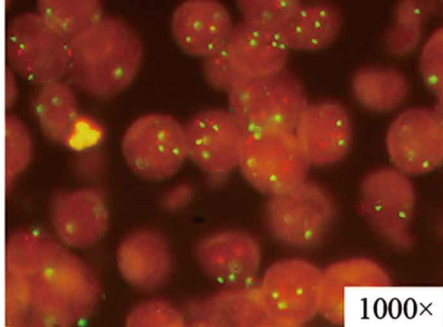

C-1

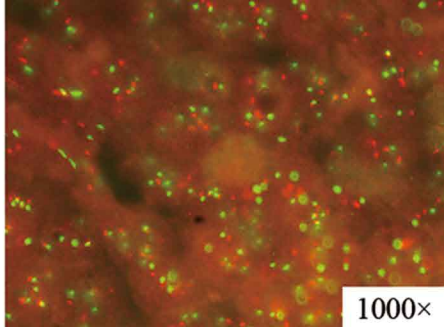

A-2

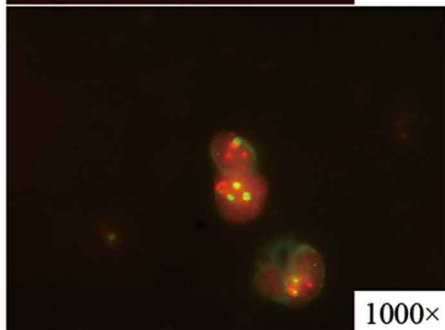

B-2

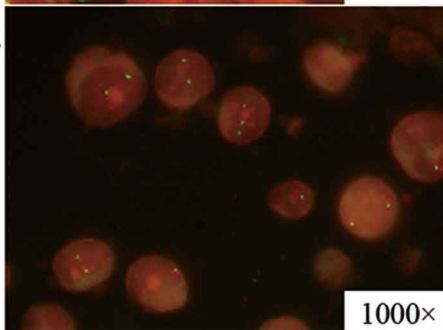

C-2

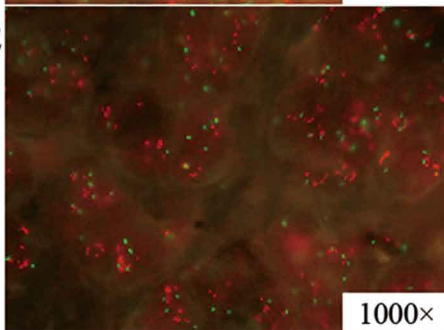

A-3

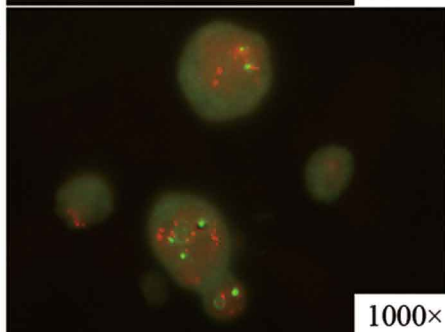

B-3

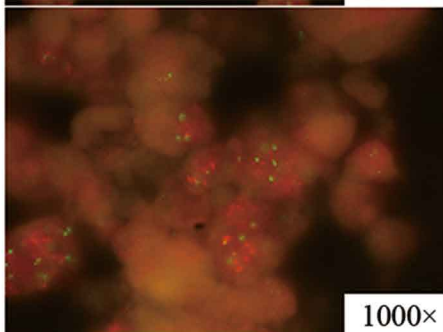

C-3

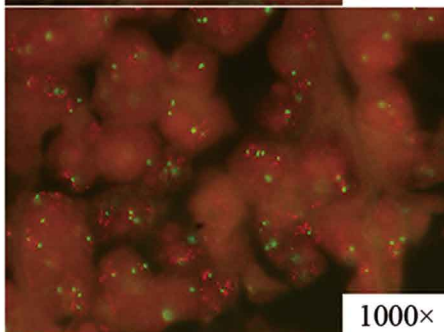

A-4

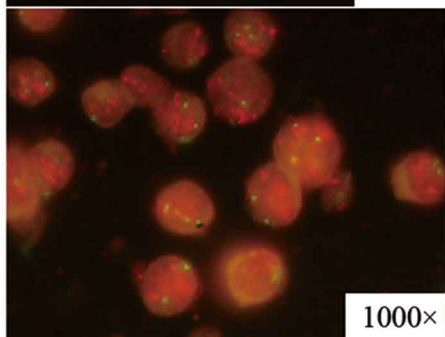

B-4

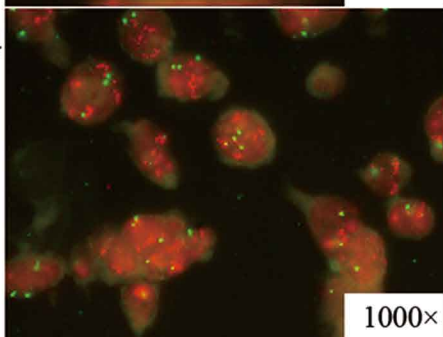

C-4

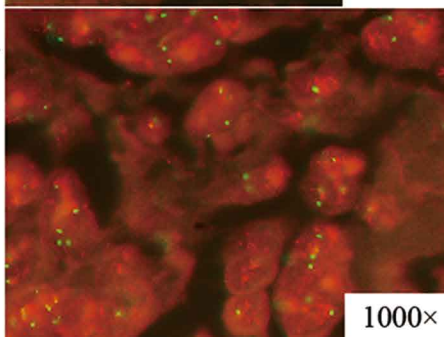

Supplement: Additional file 1: — The detailed results of HER2 gene amplification status evaluated by FISH assay. A-1 to A-4 were section of cell lines samples which were derived from MCF-7, MDA-MB-453, SKBR-3, BT474, respectively. B-1 to B-4 were section of agarose gel within cell lines samples which were derived from MCF-7, MDA-MB-453, SKBR-3, BT474, respectively. C-1 to C-4 were section of xenograft tumor samples which were derived from MCF-7, MDA-MB-453, SKBR-3, BT474, respectively. The magnification was 1000 power. (PDF 17214 kb) [file 13000_2016_537_MOESM1_ESM.pdf]

A-1

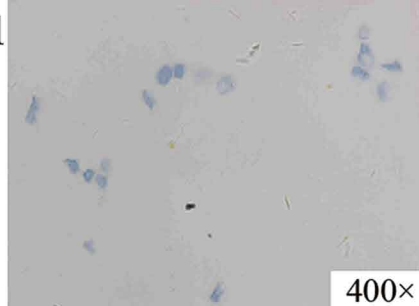

A-2

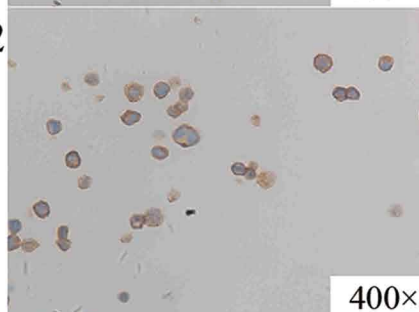

A-3

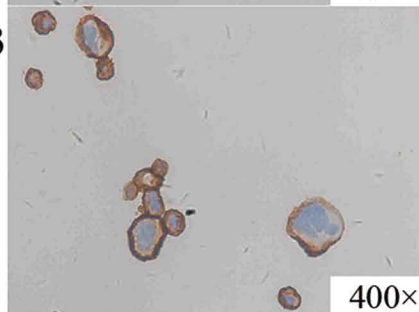

A-4

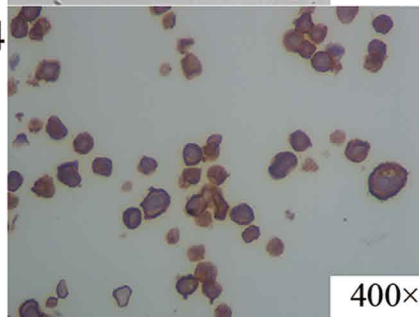

B-1

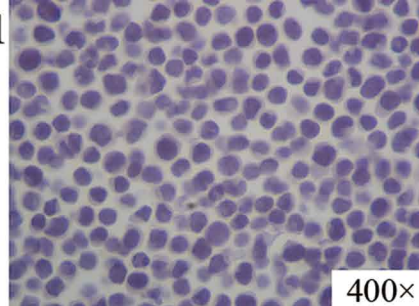

B-2

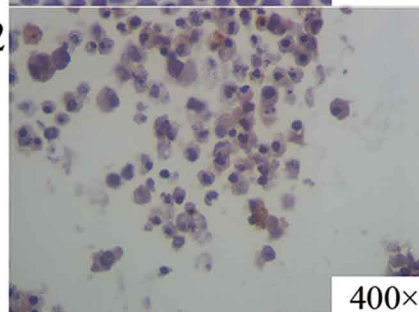

B-3

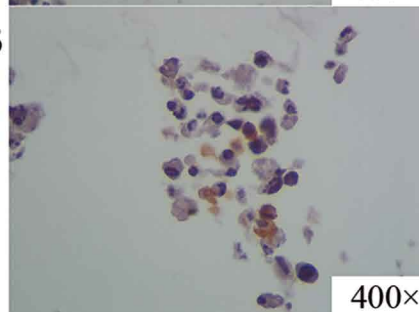

B-4

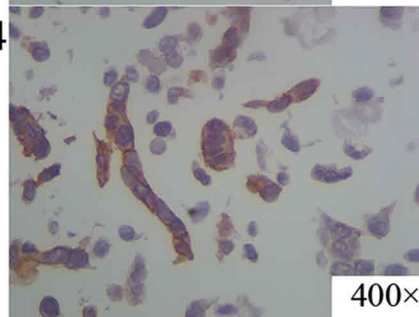

C-1

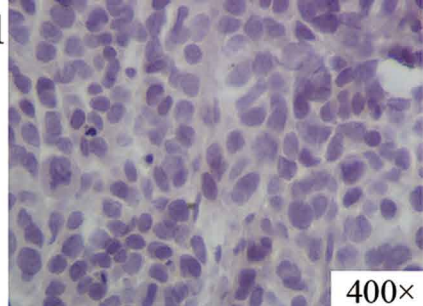

C-2

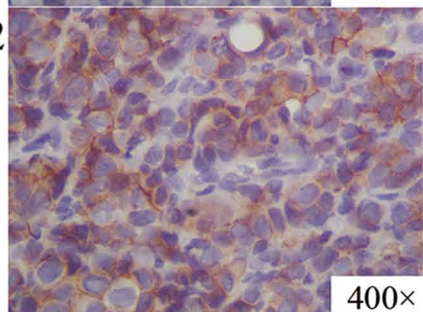

C-3

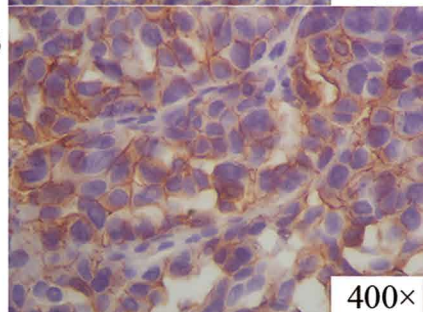

C-4

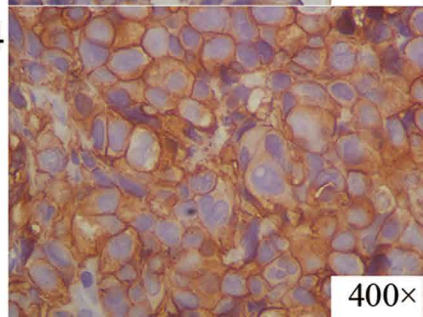

Supplement: Additional file 2: — The detailed results of HER2 protein expression status evaluated by IHC assay. A-1 to A-4 were section of cell lines samples which were derived from MCF-7, MDA-MB-453, SKBR-3, BT474, respectively. B-1 to B-4 were section of agarose gel within cell lines samples which were derived from MCF-7, MDA-MB-453, SKBR-3, BT474, respectively. C-1 to C-4 were section of xenograft tumor samples which were derived from MCF-7, MDA-MB-453, SKBR-3, BT474, respectively. The magnification was 400 power. (PDF 17387 kb) [file 13000_2016_537_MOESM2_ESM.pdf]
